# Cost‐effectiveness of implementing a multidisciplinary team approach in the management of placenta accreta spectrum

**DOI:** 10.1002/pmf2.70323

**Published:** 2026-07-01

**Authors:** Binh T. Luu, Megha Arora, Ava D. Mandelbaum, Andrew H. Chon, Tania F. Esakoff, Deirdre J. Lyell, Aaron B. Caughey

**Affiliations:** ^1^ Department of Obstetrics & Gynecology Oregon Health & Science University Portland Oregon USA; ^2^ Department of Obstetrics & Gynecology University of California Los Angeles Los Angeles California USA; ^3^ Department of Obstetrics & Gynecology Cedars Sinai Health Sciences University Los Angeles California USA; ^4^ Department of Obstetrics & Gynecology Stanford University Stanford California USA

**Keywords:** cost‐benefit analysis, maternal morbidity, multidisciplinary approach, placenta accreta spectrum, pregnancy, pregnancy complications, severe postpartum hemorrhage

## Abstract

**Objective:**

Placenta accreta spectrum (PAS) is associated with high maternal morbidity. Recent studies have shown that a multidisciplinary team (MDT) approach to managing PAS can improve outcomes. This study aimed to evaluate the cost‐effectiveness of implementing an MDT for the management of PAS.

**Methods:**

A decision‐analytic model was built using TreeAge software to compare the outcomes and cost‐effectiveness of the MDT approach versus the standard of care. The theoretical cohort included 5000 individuals in the United States with PAS annually. Maternal outcomes included severe postpartum hemorrhage (>2000 mL estimated blood loss), adjacent organ injury, intensive care unit (ICU) admission, maternal death, and hospital readmission. The cost of the MDT was estimated by assuming that there would be specific maternal‐fetal medicine (MFM) physician and nursing leadership assigned for administrative coordination and estimates of the cost of the multidisciplinary clinicians during the PAS cases. Model inputs were derived from the literature. The cost‐effectiveness threshold was $100,000/quality‐adjusted life‐year (QALY). Sensitivity analyses were performed to assess the robustness of the results.

**Results:**

An MDT approach for the management of PAS resulted in 3724 fewer severe postpartum hemorrhages, 139 fewer adjacent organ injuries, 506 fewer ICU admissions, 87 fewer hospital readmissions within 6 months, and one less maternal death compared to standard of care. When assuming a minimum baseline of 15 PAS cases per year, the MDT approach was a dominant strategy resulting in a $12.1 million cost reduction and an increase of 26 QALYs relative to standard care. One‐way sensitivity analyses on the cost of the MDT per PAS case and the number of PAS cases per medical center demonstrated that the MDT strategy remains cost effective when the cost per case was less than $9290 and when centers treat at least seven PAS cases annually.

**Conclusion:**

Implementing an MDT was cost effective and improved maternal outcomes. These findings may inform interventions that promote MDT in the management of this high‐risk condition.

## 1 INTRODUCTION

Placenta accreta spectrum (PAS) is the pathological adherence of the placenta causing maternal morbidity and mortality due to severe life‐threatening postpartum hemorrhage. PAS is subdivided into different types depending on the level of placental invasion into the uterine myometrium [1]. In the United States, the rate of PAS is estimated to be one in 804 deliveries and in all types of PAS, the primary adverse effect is severe postpartum hemorrhage which is defined as greater than or equal to 2000 mL of blood loss or blood loss with signs and symptoms of hypovolemia within 24 h after birth [2, 3, 4]. Management of the delivery of PAS involves scheduled delivery, total hysterectomy, and the use of massive transfusion protocols that have been shown to improve maternal outcomes [5].

Previous studies have shown that medical centers with a multidisciplinary team (MDT) approach for the management of PAS have improved maternal outcomes and decreased rates of maternal morbidity such as adjacent organ injury and intensive care unit (ICU) admission [6, 7, 8, 9, 10]. A recent retrospective cohort study by Luder et al. showed that the implementation of an MDT protocol was associated with less perioperative morbidity and supports that MDT approach leads to optimal management of PAS [10]. The potential downsides of an MDT approach may include subjecting patients to increased invasive procedures, which can increase maternal morbidity [7]. For example, one study found that a multidisciplinary approach led to increased surgical times, increased use of ureteral stents, and increased uterine artery embolization procedures [11]. Additionally, coordinating such an MDT will require dedicated leadership and the availability of various subspecialists, which may not be possible at many centers.

Thus, although a multidisciplinary approach for the management of PAS has been shown to improve maternal outcomes and maternal morbidity, it is unclear whether these benefits outweigh the risks and costs. This study aimed to evaluate the outcomes, costs, and cost‐effectiveness of a multidisciplinary approach for PAS versus standard of care and to determine what volumes would be required to achieve cost‐effectiveness.

## 2 MATERIALS AND METHODS

A decision‐analytic model was built using TreeAge‐Pro software to compare the outcomes and costs of an MDT approach versus standard of care for the management of placenta accreta. A decision‐tree framework was used to simulate the clinical pathways of PAS, calculating expected values for costs and health outcomes based on transition probabilities at each branch. The model assessed adverse maternal outcomes (e.g., severe postpartum hemorrhage, ICU admission), costs, and quality‐adjusted life‐years (QALYs) based on an MDT or standard of care approach. A QALY is used to capture the quality of life an individual experiences in a particular health state and is used to measure effectiveness [12]. The cost‐effectiveness threshold for an intervention is deemed cost effective depending on a society's willingness to pay, which is generally set at $100,000 per QALY in the United States [13]. Since no human subjects were involved in the creation of this model, this study was deemed exempt from the Institutional Review Board at the Oregon Health & Science University.

For this analysis, we assessed outcomes for a theoretical cohort consisting of 5000 women in the United States with PAS annually [2]. Our model began with pregnant women with confirmed PAS. The first branching node was whether the management approach was done by an MDT or standard of care. Subsequent nodes included outcomes such as severe maternal hemorrhage, adjacent organ injury, ICU admission, hospital readmission, and maternal death. These nodes represent likely outcomes under average clinical conditions, providing a benchmark against which all subsequent variations are measured. This base case used in the model is just one iteration of a much wider spectrum of possibilities. All model inputs were derived from the literature (Table 1). The perspective of this analysis was conducted from a societal perspective.

**TABLE 1:** Model inputs.

Variable	Value	Range in sensitivity analyses	References
Probabilities			
Severe postpartum hemorrhage^a^ cases (multidisciplinary team approach)	0.185	0.0–0.41	Licon et al. [8]
Severe postpartum hemorrhage^a^ cases (standard of care)	0.929	0.83–1.0	O'Brien et al. [14]
ICU admissions (multidisciplinary team approach)	0.088	0.01–0.16	Flores‐Mendoza et al. [15]
ICU admissions (standard of care)	0.355	0.17–0.54	Eller et al. [9]
Adjacent organ injury cases (multidisciplinary team approach)	0.063	0.0–0.15	Eller et al. [9]
Adjacent organ injury cases (standard of care)	0.081	0.0–0.18	Eller et al. [9]
Maternal deaths (multidisciplinary team approach)	0.016	0.0–0.06	Eller et al. [9]
Maternal deaths (standard of care)	0.017	0.0–0.07	Shamshirsaz et al. [7]
Readmissions within 6 months (multidisciplinary team approach)	0.101	0.0–0.2	Eller et al. [9]
Readmissions within 6 months (standard of care)	0.129	0.0–0.26	Eller et al. [9]
Cost (2024 USD)			
Severe maternal hemorrhage^b^	$4688	$1100–$8276	Shander et al
ICU admission^c^	$37,793	$15,966–$120,092	Wymer et al. [16, 17]
Adjacent organ injury	$20,891	$5785–$43,516	Venkatesh et al. [18]
Hospital readmission	$23,009	$5400–$40,618	Phibbs et al. [19]
Maternal death^d^	$1,727,044	$405,326–$3,048,762	Flores‐Mendoza et al. [15]
Cost of multidisciplinary team per case^e^	$6366	$1494–$11,239	US Bureau of Labor Statistics [20]
Cost of multidisciplinary team annually	$95,504	$19,363–$145,646	US Bureau of Labor Statistics [20]
Cost of standard of care per case^f^	$4066	$954–$7178	US Bureau of Labor Statistics [20]
Utilities			
Severe maternal hemorrhage^a^	0.32	0.17–0.47	Joshi et al. [21]
ICU admission	0.49	0.34–0.64	Joshi et al. [21]
Maternal death	0	0.62–0.92	Joshi et al. [21]
Adjacent organ injury	0.77	27.3–56.7	Venkatesh et al. [18]
Maternal life expectancy^g^ (years)	42	0.17–0.47	Garmi et al. [22]

Abbreviations: FTE, full‐time equivalent; ICU, intensive care unit; OBGYN, obstetrics and gynecology; pRBC, packed red blood cells; USD; US dollars.

^a^ Defined as > 2000 mL estimated blood loss.

^b^ Cost derived from the cost of four transfusion units of packed red blood cells (pRBC).

^c^ Cost derived from average days of ICU stay (2.5 days) multiplied by cost per day ($15,117) [16].

^d^ Cost in months/year.

^e^ Estimated by summing 10% FTE of a medical director, 20% FTE of a nurse coordinator, and standard work relative value units (wRVUs) of subspecialists for a 4‐h case.

^f^ Estimated by summing wRVUs of OBGYN generalist, surgical assistant, and anesthesiologist for a 4‐h case.

^g^ Maternal life expectancy after delivery.

Cost data were derived from the literature and inflated to 2024 US dollars using the medical component of the Consumer Price Index [23]. In our model, the MDT approach includes structured coordination among subspecialists, preoperative planning, and organized resource allocation, whereas standard care represents management without a formally coordinated MDT structure or systematic preoperative planning. We estimated that the administrative costs would include 10% full‐time equivalent (FTE) of a medical director and 20% FTE of a nurse coordinator. The estimate of MDT cost also included estimates of the cost of the clinicians during the PAS cases. This estimate was based on standard work relative value units (wRVUs) and current US national conversion factors for a 4‐h surgical case. The subspecialists incorporated in our MDT cost estimate include MFM, gynecologic oncology, and anesthesiology. The total annual cost was estimated by combining the annual administrative cost plus the per case cost times the average annual number of PAS a medical center could potentially care for. This total cost could then be divided by the number of PAS cases to generate a cost per case, which could then be varied in sensitivity analysis. As the standard approach for the management of PAS includes scheduling delivery between 34 and 35 weeks and these cases occur in environments that already have on‐call providers, costs for a 24/7 call schedule were not included [24]. The cost of standard of care included the professional fees for an obstetrics and gynecology (OBGYN) generalist, a surgical assistant, and an anesthesiologist. Cost data for severe postpartum hemorrhage, adjacent organ injury, ICU admission, hospital readmission, and maternal death are shown in Table 1.

Maternal QALYs in our analysis were evaluated based on utilities applied to life expectancy (Table 1). Utilities are a proxy for health, with 0 representing death and 1 representing perfect health. QALYs were estimated by applying utilities found in the literature to life expectancy discounted at a rate of 3% [25]. We calculated costs and QALYs to determine the incremental cost‐effectiveness ratio (ICER), which is the cost of the strategy per additional QALY gained. The ICER is calculated by dividing the difference in cost by the difference in QALYs between the two management approaches. A willingness‐to‐pay (WTP) threshold of $100,000 per QALY or less was considered cost effective. In cost‐effective analysis, a strategy is considered dominant if it is associated with lower costs and higher QALYs.

We performed one‐way sensitivity analyses on all probabilities, costs, and utilities to assess the robustness of the model inputs. We ranged the value of the number of PAS cases to look for threshold values. A threshold value is a value in which the optimal strategy changes at the specified WTP value. Indicator variables were used to simultaneously vary multiple probabilities at once. We created a tornado diagram to determine which inputs had the most significant effect on outcomes.

Finally, a Monte Carlo simulation was performed to evaluate the multivariable changes in all model inputs across their respective distributions. The simulation was run 100,000 times. For the Monte Carlo analysis, a beta distribution was used to assess probability and utility estimates. Cost estimates were assessed with a gamma distribution with the right skew accounting for outliers in the upper‐bound range of medical costs and a triangular distribution was used for durations and life expectancies. If the standard deviation was unavailable in the literature, we estimated a wide range to consider the minimum and maximum extremes. We reported the Monte Carlo results with a 95% confidence ellipse and calculated the proportion of the time each strategy is cost effective.

## 3 RESULTS

In our theoretical cohort of 5000 women with PAS, the MDT strategy was associated with 3724 fewer cases of severe postpartum hemorrhage, 139 fewer ICU admissions, 108 fewer cases of adjacent organ injury, 87 fewer cases of hospital readmissions within 6 months, and one fewer maternal death compared to standard of care (Table 2). When assuming a minimum baseline of 15 PAS cases per year, the MDT approach was a dominant strategy resulting in a $12.1 million cost reduction and an increase of 26 QALYs relative to standard care.

**TABLE 2:** Maternal outcomes, cost, and effectiveness associated with the presence of multidisciplinary team versus standard of care in the management of PAS.

	Multidisciplinary team approach	Standard of care	Difference
Severe postpartum hemorrhage^a^ (cases)	926	4650	−3724
ICU admissions	506	645	−139
Adjacent organ injury (cases)	440	548	−108
Maternal deaths	80	81	−1
Readmissions within 6 months	316	403	−87
Cost ($USD)	195,161,750	207,224,650	−12,062,900
Effectiveness (QALYs)	116,584	116,558	26
Strategy	Dominant	Dominated	

Abbreviations: ICU, intensive care unit; PAS, placenta accreta spectrum; QALY, quality‐adjusted life‐year; USD, US dollars.

^a^ Defined as >2000 mL estimated blood loss.

**FIGURE 1:**
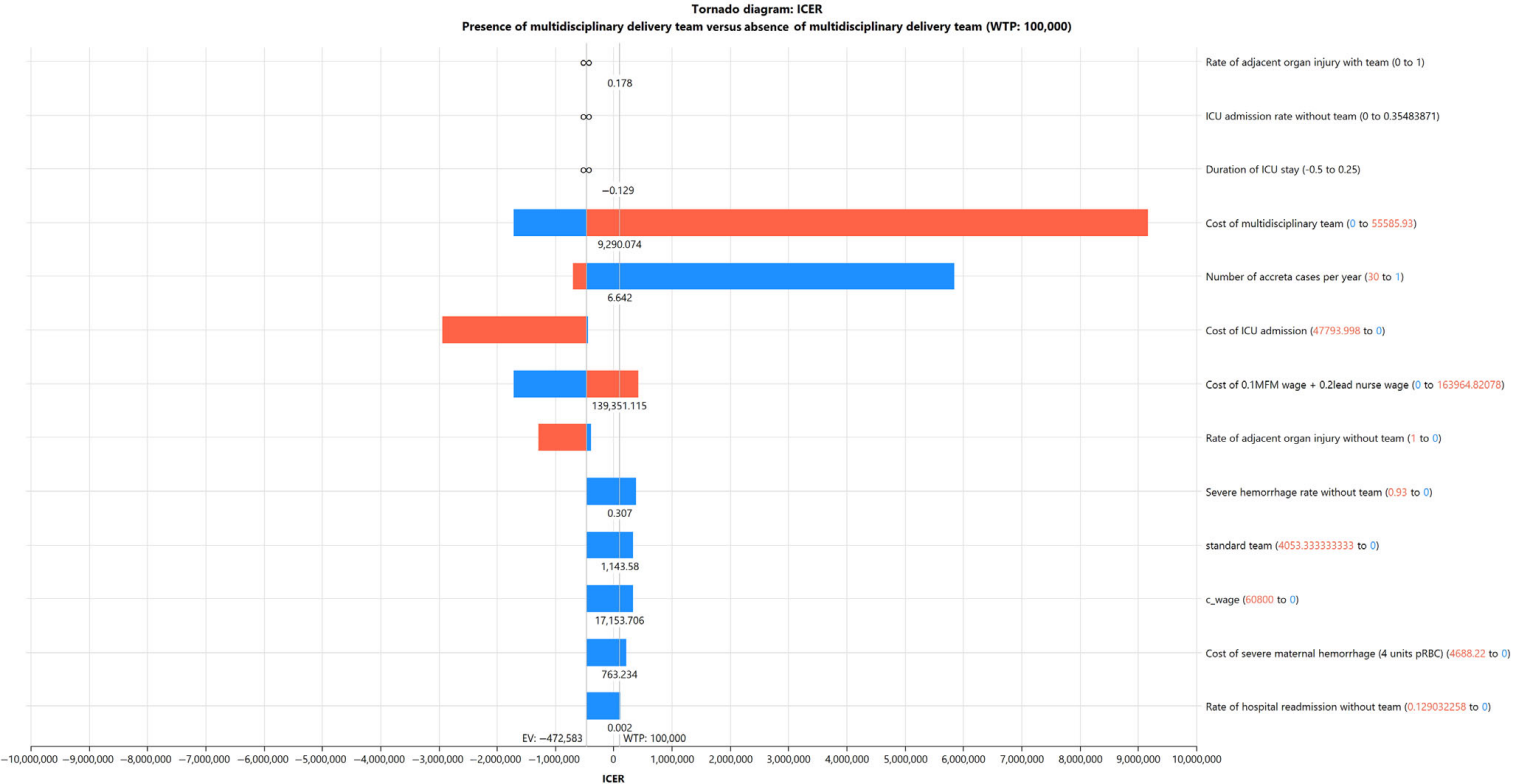
ICER tornado diagram. Each horizontal column represents the changes in the incremental cost‐effectiveness ratio (ICER) while varying the input probability or cost listed beside the column. The gray vertical line represents the willingness‐to‐pay (WTP) threshold at $100,000 US dollars. Probabilities or costs that crossed the gray WTP threshold in the figure represent a variable that changes cost‐effectiveness when varied. Variables that had a ∞ symbol were found to be strong drivers in the model and were further evaluated in univariate sensitivity analyses. EV, expected value; ICU, intensive care unit; MFM, maternal‐fetal medicine; pRBC, packed red blood cells.

**FIGURE 2:**
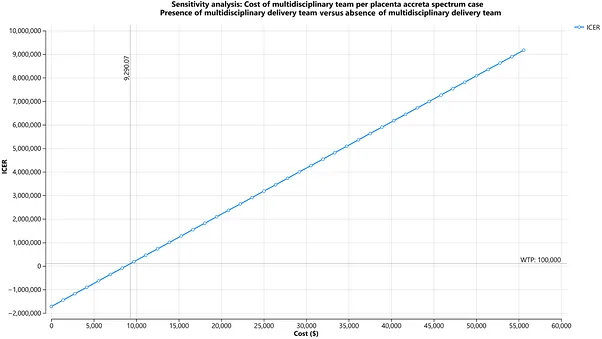
One‐way sensitivity analysis varying the cost of the multidisciplinary team per PAS case. A multidisciplinary team that costs less than $9290 per case resulted in the incremental cost‐effectiveness ratio (ICER) falling below the willingness‐to‐pay (WTP) threshold, making it the dominant strategy. PAS, placenta accreta spectrum.

**FIGURE 3:**
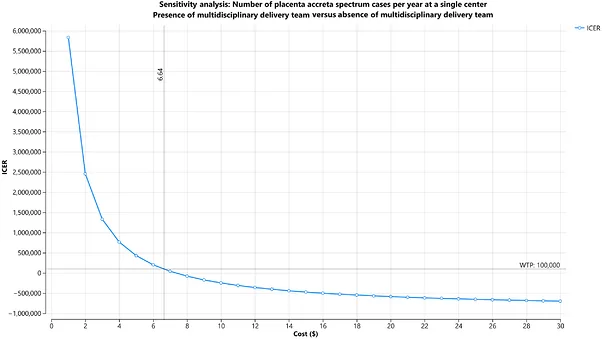
One‐way sensitivity analysis varying the number of PAS cases a medical center treat. Managing greater than seven PAS cases annually resulted in the incremental cost‐effectiveness ratio (ICER) falling below the willingness‐to‐pay (WTP) threshold. PAS, placenta accreta spectrum.

The number of PAS cases per year a medical center treats and the cost of the MDT had a significant impact on the ICER in the tornado diagram (Figure 1). The cost of the MDT per PAS case had the most influence on the cost‐effectiveness of the multidisciplinary versus standard of care strategies. Thus, we performed a one‐way sensitivity analysis varying the cost of the MDT per PAS case. This analysis revealed that the ICER of the MDT approach remained below the WTP threshold when the total cost per case was less than $9290 (Figure 2). A one‐way sensitivity analysis was also done varying the number of PAS cases per year a center treats. This analysis revealed that the ICER of the MDT approach remained below the WTP threshold when centers treat at least seven PAS cases annually (Figure 3).

**FIGURE 4:**
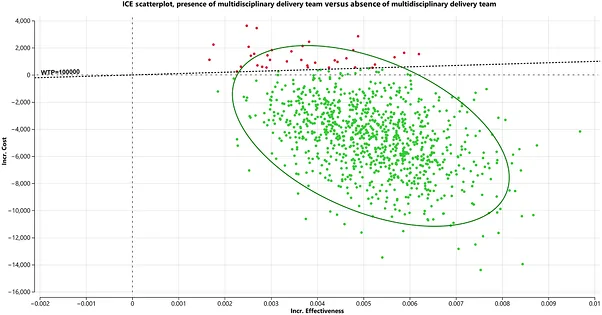
Multivariate sensitivity analysis. This Monte Carlo simulation displays the outcomes of the 100,000 trials of the simulation. The dashed line indicates the willingness‐to‐pay (WTP) threshold, which was set at $100,000. Each dot represents the result of a single trial. The ellipse represents the 95% confidence ellipse. The multidisciplinary team strategy was the dominant strategy in 96.5% of the trials.

Probabilities or costs that crossed the WTP threshold in Figure 1 represent variables that change cost‐effectiveness when varied. Variables that had an ∞ symbol were also found to be strong drivers in the model and were further evaluated in univariate sensitivity analyses. Varying duration of ICU stay and the probabilities of acute organ injury in the presence and absence of the MDT were found to be other strong drivers in the model. A vertical dashed line indicates a significant transition in cost‐effectiveness. For the univariate analysis of acute organ injury with MDT, the MDT approach was cost saving until the rate of an acute organ injury occurring was greater than 70% at which point this strategy was more expensive and resulted in lower QALYs. For the univariate analysis of the probability of ICU admission, the absence of the MDT showed a dominant strategy up to 8%, meaning that standard of care was cost saving if the rate of ICU admission was less than 8%. For the univariate analysis of ICU duration, ICU stays greater than a day showed that the MDT approach results in decreased QALYs and increased costs, turning this from a dominant to a dominated strategy. Once variability was incorporated into our model inputs via Monte Carlo simulation, we found that the MDT strategy was dominant in 96.5% of samples (Figure 4).

## 4 DISCUSSION

### 4.1 Principal findings

This study demonstrated that an MDT approach for the treatment of PAS was cost effective and was associated with a lower number of cases of severe postpartum hemorrhage, ICU admission, adjacent organ injury, maternal hospital readmission, and maternal death. For medical centers that treated greater than seven PAS cases a year, the MDT approach was also the cost‐saving strategy. Relative to the median salaries for one MFM physician and one nurse leader and the professional fees of subspecialists, medical centers that have an MDT for the treatment of PAS could save an estimated $12.1 million of costs annually in the United States. The MDT approach also remained cost effective when varying the cost of the team per case up to $9290.

### 4.2 Results in the context of what is known

This analysis was conducted from a societal perspective, capturing both direct healthcare costs and broader downstream economic consequences associated with PAS management. Under this framework, we incorporated not only the immediate costs of MDT implementation and index hospitalization, but also the costs associated with complications, readmissions, and long‐term morbidity that extend beyond the treating institution. By accounting for these downstream effects, our model reflects the full economic impact of care delivery on patients, payers, and the healthcare system as a whole. This perspective is particularly relevant for PAS, where adverse outcomes carry substantial long‐term resource utilization and societal burden. As such, while MDT programs may require upfront institutional investment, the overall cost savings and health benefits accrue at the population level, supporting the value of MDT implementation as a high‐impact, system‐wide intervention.

The economic value of MDT implementation extends beyond the index hospitalization. While upfront investments in personnel, infrastructure, and care coordination are required, these costs are offset by reductions in high‐acuity complications and downstream healthcare utilization, including reoperation, prolonged hospitalization, and long‐term morbidity. As PAS incidence continues to rise, the cumulative societal burden of suboptimal management will increase, further amplifying the long‐term cost benefits of MDT‐coordinated care models. However, a key challenge lies in the misalignment of financial incentives: although MDTs generate clear societal savings, these benefits are often realized by payers rather than the institutions that bear the implementation costs. This disconnect underscores the need for alternative funding strategies, such as state‐level investment, bundled payments, or enhanced reimbursement for complex obstetric care, particularly within designated centers of excellence.

Past studies have similarly shown better maternal outcomes for PAS with the presence of an MDT [6, 7, 8, 9, 11, 15]. In one retrospective cohort study, an MDT approach for the management of PAS was associated with lower rates of maternal morbidity when compared to standard of care [9]. Studies that have compared an MDT approach versus standard of care for PAS cited that the additional experience and expertise from an MDT are the key factors for differences in outcomes because these teams develop a greater expertise for PAS management and can quickly act accordingly.

Sustainability of MDT programs remains a critical concern. Currently, these teams are supported through a combination of hospital system investment, departmental resources, and professional billing, a structure that often undervalues the extensive interdisciplinary coordination required for PAS management. Activities such as preoperative planning, multidisciplinary case conferences, and protocol development are rarely reimbursed despite their central role in improving outcomes. However, one study has shown that an MDT program for PAS was associated with a reduction in resource use and variability [26]. Given the demonstrated societal and resource benefits, MDTs should ultimately be supported through broader healthcare system investment, with consideration for formal designation and funding of specialized centers of excellence.

In this context, regionalization of PAS care may represent a pragmatic strategy to optimize both outcomes and resource utilization. Prior studies have demonstrated improved maternal outcomes when PAS cases are concentrated in high‐volume centers with specialized expertise. Our findings further support this model by demonstrating that cost savings are most pronounced in institutions managing higher annual case volumes. Centralizing care in designated centers of excellence may facilitate efficient deployment of MDT resources, improve team proficiency, and enhance care coordination. However, such an approach must also consider geographic access and potential disparities in care delivery, particularly for patients in rural or underserved regions.

The inclusion of other subspecialists, such as interventional radiology (IR) and urology, was considered in the MDT framework. However, the role of IR in PAS management remains variable and incompletely defined. While some literature suggests that IR may improve outcomes [27, 28], the evidence remains mixed; for instance, some findings diverge from traditional benchmarks, while other studies suggest that robust MDT surgical management may actually reduce the need for endovascular techniques altogether [29, 30]. Due to this uncertainty, we did not include IR in our base MDT cost estimate. Similarly, the urologic injury rates are well documented and have been shown to be a significant complication of PAS management [31, 32]. However, urology was not included in our MDT cost estimate as the regular use of ureteral stents is unclear and has been associated with higher rates of urinary irritation and hematuria [33]. Nevertheless, our sensitivity analysis demonstrated that the MDT approach remains cost effective up to a cost threshold that could comfortably incorporate IR and urology coverage, providing flexibility for institutions where IR or urology is a routine component of PAS management.

Our findings also support the growing body of literature advocating for the regionalization of PAS care [34]. Concentrating care in high‐volume centers allows for greater team proficiency, improved outcomes, and maximization of cost savings, consistent with prior studies demonstrating volume–outcome relationships. However, regionalization must be balanced against considerations of geographic access and potential disparities in care, particularly for patients in underserved areas.

### 4.3 Strengths and limitations

Considering the methodological limitations of cost‐effectiveness analyses, this model is liable to uncertainty in the inputs for the selected probabilities, costs, and utilities. To minimize this drawback, most of our model inputs were cited from national data or large population cohort studies from the United States. However, our inputs that involved the MDT strategy were derived from smaller studies with limited data and may be subject to variation. However, we varied such inputs over a wider range and incorporated their distributions in the Monte Carlo simulation to examine such uncertainty further. We believe that our analysis provides valuable insight into the cost‐benefit of implementing an MDT approach for the management of PAS.

An important consideration in our cost assumptions was the availability of subspecialty 24/7 support and coverage. We did not include additional costs for 24/7 on‐call coverage, as these services are typically already embedded within tertiary care centers. As the other multidisciplinary members of the team typically do not perform high‐risk pregnancy procedures, we included the professional reimbursement for their specialty services. However, in lower‐resource or community settings where such coverage is not routinely available, the incremental costs of ensuring continuous subspecialty access could meaningfully impact cost‐effectiveness. In these contexts, regionalization of PAS care may represent a more economically and clinically viable strategy.

Our analysis should also be interpreted in the context of several limitations. The generalizability of our findings may be influenced by assumptions regarding national PAS case volume, including our estimate of 5000 annual cases, as well as variability in institutional resources, payer structures, and clinical practice patterns across health systems. Differences in regional costs, access to subspecialty care, and institutional infrastructure may impact both the feasibility and cost‐effectiveness of MDT implementation. Future studies should evaluate these factors in diverse healthcare settings to better define context‐specific strategies for optimizing PAS care. While PAS cases continue to increase annually in the United States, the implementation of MDTs across all birth hospitals is neither feasible nor economically sound. Our analysis supports the growing body of evidence suggesting that centralization of PAS care is essential [34, 35]. By concentrating PAS cases in high‐volume centers, health systems can optimize and maximize the cost‐saving potential identified in our study.

Finally, with respect to economic evaluation methodology, we recognize that when one strategy is both more effective and less costly, it is considered dominant and the calculation of an ICER is not appropriate. However, in our sensitivity analyses where non‐dominance was observed, ICERs remained a useful measure to quantify the cost per quality‐adjusted life year gained. This approach provides a more nuanced assessment of value by capturing the trade‐offs between increased upfront investment and meaningful improvements in maternal health outcomes.

## 5 CONCLUSIONS

In summary, MDT‐based care for PAS represents a high‐value intervention that improves clinical outcomes while remaining cost effective. The MDT approach for the management of PAS was cost saving and reduced maternal morbidity. However, the cost per PAS case and the number of cases a medical center cares for each year impacts the cost‐effectiveness of the MDT strategy. Our results indicate that such an MDT would be a cost‐effective and cost‐saving approach for medical centers that care for and deliver seven cases of PAS a year. The MDT approach was also cost effective when the cost per PAS case is below $9290. Medical centers that care for fewer than seven PAS cases per year would still gain clinical benefit but may not be willing to invest in such a team. Such centers should consider the tradeoffs of caring for such individuals themselves versus referring or transferring them to other, higher‐volume centers. Given the benefits of MDT implementation extend beyond individual institutions to society at large, future efforts should focus on aligning financial incentives, developing sustainable funding mechanisms, and promoting regionalized systems of care to support the delivery of coordinated, multidisciplinary management for this increasingly prevalent and high‐risk condition. (Supporting Information)

## CONFLICT OF INTEREST STATEMENT

The authors declare no conflicts of interest.

## FUNDING INFORMATION

The authors received no specific funding for this work.

## Supporting information



Supporting information
