# Supplementary material for: Cost‐effectiveness of implementing a multidisciplinary team approach in the management of placenta accreta spectrum
Source: Pregnancy (Hoboken). 2026 Jul 1;2(4):e70323. doi: 10.1002/pmf2.70323 (PMC13344407; doi:10.1002/pmf2.70323)
Supplement: Supplementary file 1 [file PMF2-2-e70323-s001.docx]

For economic modeling, TreeAge Pro software uses structured pathways to simulate patient outcomes. The foundational modeling approach utilizes Decision Trees to map out clinical scenarios, starting from an initial decision point and branching into various possible events, such as treatment success, complications, or side effects. By assigning probabilities to each branch and attaching specific costs and health outcomes to the final "terminal nodes," the model calculates the weighted average—or expected value—for each strategy. This allows clinicians to move beyond individual case studies and project the impact of a clinical decision across a standardized population.

The primary metric for measuring health outcomes in these models is the Quality-Adjusted Life Year (QALY). Unlike simple survival rates, QALYs account for the quality of life by multiplying the time spent in a health state by a "utility" weight, where 1.0 represents perfect health and 0 represents death. When comparing a new intervention to the current standard of care, we calculate the Incremental Cost-Effectiveness Ratio (ICER). This ratio represents the additional cost required to gain one extra QALY. In formal terms, the formula is defined as:

$$ICER= \frac{New Cost-Standard Cost}{New Effectiveness-Standard Effectiveness}$$

For a new intervention to be considered "cost-effective" in most healthcare systems, its ICER must fall below a specific Willingness-to-Pay (WTP) threshold, which serves as the maximum budget a society is willing to spend for a single QALY gained.

It is vital to distinguish between a strategy that is cost-saving and one that is merely cost-effective. A cost-saving intervention is both more effective and less expensive than the alternative; in economic terms, this strategy "dominates" the competition and is plotted in the favorable bottom-right quadrant of the Cost-Effectiveness (CE) Plane. Conversely, a cost-effective intervention is usually more effective but also more expensive. It is only considered a viable choice if the extra health gain justifies the extra spend—essentially, if the ICER is lower than the WTP threshold. Graphically, TreeAge displays this through the Cost-Effectiveness Frontier, a line connecting the most efficient strategies. Any treatment located above or to the left of this frontier is considered "dominated" and is generally excluded from clinical recommendations.

Finally, clinicians must account for the uncertainty inherent in clinical data using Probabilistic Sensitivity Analysis (PSA). Instead of a single fixed result, TreeAge generates an ICE Scatterplot, which appears as a "cloud" of results based on thousands of simulations that vary the input data. This cloud visualizes the probability that an intervention will remain cost-effective even if parameters like drug costs or complication rates fluctuate. If the majority of the points in the scatterplot fall below the diagonal WTP threshold line, the clinician can have high confidence that the intervention represents a value-based choice for their patient population.

References:

1. Detsky AS, Naglie G, Krahn MD, Redelmeier DA, Naimark D. Primer on medical decision analysis: Part 2—building a tree. *Med Decis Making*. 1997;17(2):126-135. doi:10.1177/0272989X9701700202.

2. Lee A, Joynt GM, Ho AMH, et al. Tips for teachers of evidence-based medicine: making sense of decision analysis using a decision tree. *J Gen Intern Med*. 2009;24(5):642-648. doi:10.1007/s11606-009-0918-8.

3. Briggs AH, Ades AE, Price MJ. Probabilistic sensitivity analysis for decision trees with multiple branches: use of the Dirichlet distribution in a Bayesian framework. *Med Decis Making*. 2003;23(4):341-350. doi:10.1177/0272989X03255922.

4. Rubrichi S, Rognoni C, Sacchi L, et al. Graphical representation of life paths to better convey results of decision models to patients. *Med Decis Making*. 2015;35(3):398-402. doi:10.1177/0272989X14565822.

5. Banerjee M, Reynolds E, Andersson HB, Nallamothu B. Tree-based analysis: a practical approach to create clinical decision making tools. *Circ Cardiovasc Qual Outcomes*. 2019;12(5):e004879. doi:10.1161/CIRCOUTCOMES.118.004879.
